# Differences in brain activity between normal and diabetic rats under isoflurane anesthesia: a resting-state functional MRI study

**DOI:** 10.1186/s12880-022-00867-6

**Published:** 2022-08-04

**Authors:** Sheng-Min Huang, Chun-Yi Wu, Yu-Hsin Lin, Hsin-Hua Hsieh, Hui-Chieh Yang, Shao-Chieh Chiu, Shin-Lei Peng

**Affiliations:** 1 Institute of Biomedical Engineering and Nanomedicine, National Health Research Institutes, Miaoli, Taiwan; 2 Department of Biomedical Imaging and Radiological Sciences, Taipei Branch, National Yang Ming Chiao Tung University, Taipei, Taiwan; 3 Department of Pharmacy, Taipei Branch, National Yang Ming Chiao Tung University, Taipei, Taiwan; 4 Department of Medical Research, China Medical University Hospital, China Medical University, Taichung, Taiwan; 5 Department of Biomedical Imaging and Radiological Science, China Medical University, Taichung, Taiwan; 6 Center for Advanced Molecular Imaging and Translation, Chang Gung Memorial Hospital, Taoyuan, Taiwan; 7 Neuroscience and Brain Disease Center, China Medical University, Taichung, Taiwan

**Keywords:** Anesthesia, fALFF, Hyperglycemia, Neural, Streptozotocin (STZ)

## Abstract

**Background:**

Altered neural activity based on the fractional amplitude of low-frequency fluctuations (fALFF) has been reported in patients with diabetes. However, whether fALFF can differentiate healthy controls from diabetic animals under anesthesia remains unclear. The study aimed to elucidate the changes in fALFF in a rat model of diabetes under isoflurane anesthesia.

**Methods:**

The first group of rats (n = 5) received a single intraperitoneal injection of 70 mg/kg streptozotocin (STZ) to cause the development of diabetes. The second group of rats (n = 7) received a single intraperitoneal injection of the same volume of solvent. Resting-state functional magnetic resonance imaging was used to assess brain activity at 4 weeks after STZ or solvent administration.

**Results:**

Compared to the healthy control animals, rats with diabetes showed significantly decreased fALFF in various brain regions, including the cingulate cortex, somatosensory cortex, insula, and striatum (all P < 0.05). The decreased fALFF suggests the aberrant neural activities in the diabetic rats. No regions were detected in which the control group had a lower fALFF than that in the diabetes group.

**Conclusions:**

The results of this study demonstrated that the fALFF could be used to differentiate healthy controls from diabetic animals, providing meaningful information regarding the neurological pathophysiology of diabetes in animal models.

## Introduction

Diabetes is a chronic metabolic disease characterized by a relative or absolute lack of insulin, resulting in hyperglycemia. An estimated 463 million people worldwide have diabetes, with rates continuing to rise [1]. Although diabetes is not immediately fatal, it deteriorates the patient quality of life. Over time, diabetes can negatively affect the heart, blood vessels, eyes, kidneys, and nerves. Furthermore, as diabetes is a systemic disease affecting several organs, including the brain, recent research has also focused on diabetes-related brain complications [2, 3]. Diabetes also increases the risk of stroke [4] and Alzheimer’s disease [5]. Therefore, improving our understanding of the physiological characteristics of brain function in individuals with diabetes may shed light on the pathophysiological aspects underlying diabetes-related brain injuries.

Resting-state functional magnetic resonance imaging (rs-fMRI) is a useful tool for assessing spontaneous neural activity. The fractional amplitude of low-frequency fluctuation (fALFF) of the blood oxygen level- dependent (BOLD) signals, as an rs-fMRI analysis algorithm, is a powerful index for globally reflecting spontaneous neuronal activity and physiological states [6]. Unlike functional connectivity analyses, which focus on the relationships among different regions, fALFF measures the intensity of neural activity at the single-region level. Due to this unique characteristic, fALFF has been used as an effective method in assessing the spontaneous neuronal activity of diseases, and Zhang et al. also reported aberrant fALFF in many brain regions in patients with diabetes, the findings of which are suggestive of diabetes-related brain dysfunction [7]. However, one overlooked aspect in the aforementioned study [7], and even in other diabetes rs-fMRI studies [8, 9], is that they have covered life stages from middle to old age. However, fALFF variations across older individuals may be related to other variations in physiology, such as hypertension [10] and obesity [11]. Factoring out such confounders can extract the key brain regions which are associated with diabetes, rather than its clinical comorbidities.

By intraperitoneal injection of a single high-dose or multiple low-doses of streptozotocin (STZ), experimental diabetic rats can develop cerebral dysfunctions similar to those observed in patients with diabetes [12] with low cost. Moreover, animal experiments offer an advantage in that we can ignore other disease-related pathologies, such as hypertension and obesity, thereby reflecting the diabetes-related brain alterations alone. Therefore, there are growing applications for animal models in diabetes research, and they are helpful for understanding diabetes pathogenesis and developing new treatments [13, 14]. fALFF is not a new approach in the animal studies, and it is a useful tool to evaluate significant brain changes related to ischemic stroke in the rats [15]. However, no information is currently available regarding the application of fALFF in a rat model of diabetes.

The objective of this study was to elucidate the changes in fALFF [6] in a rat model of diabetes under isoflurane anesthesia for the first time. We hypothesized that the fALFF method is sufficiently sensitive to differentiate healthy controls from diabetic animals, even under anesthesia.

## Methods and materials

### Animal preparation

This study used a total of 13 male Sprague–Dawley rats (7 weeks old, 245–295 g), and seven and six of which were used for the control and diabetes groups, respectively. The animals were kept under standard conditions (lights on from 6 AM to 6 PM) and provide a standard rodent diet and water ad libitum. All animal experiments were approved by the Institutional Animal Care and Use Committee of the China Medical University (CMUIACUC-2018-073) and were carried out in accordance with the approved guidelines.

Diabetes was induced in rats in the diabetes group at 8 weeks of age by a single intraperitoneal injection of 70 mg/kg of streptozotocin (STZ, Sigma Chemical Co., St. Louis, MO) dissolved in 0.1 M sodium citrate buffer, which caused specific necrosis of the pancreatic β–cells and resembles pathological manifestations of human type I diabetes [16]. Animals in the control group received a single intraperitoneal injection of the same volume of solvent. After STZ or citrate buffer administration, the animals were monitored weekly by measuring body weight and non-fasting plasma glucose concentration. The plasma glucose concentration was determined using a glucometer (Accu-Chek, Basel, Switzerland). Animals with non-fast plasma glucose concentrations > 250 mg/dL were considered diabetic and used for the following study. One animal died 2 weeks after STZ administration, leaving five rats in the diabetes group. All animals underwent MRI scans 4 weeks after STZ or solvent administration.

### MRI experiments

All MRI experiments were conducted using a 7 T animal MRI scanner (Bruker ClinScan 70/30, Germany) with a gradient strength of 630 mT/m. During the MRI scans, the animals were anesthetized using medical air (1.0 L/min) with isoflurane (1.0–1.5%). Their body temperatures were maintained using a warm water circulation system and measured using a rectal probe. Physiological parameters, including heart rate and breath rate, were recorded during the experiments (SA instruments Inc., NY, USA). Scout images along three orientations were acquired by using the T1-weighted sequence. For BOLD rs-fMRI images, 300 consecutive volumes with 11 coronal slices were acquired with the following parameters: field of view (FOV) = 30 × 30 mm^2^, matrix size = 64 × 64, nine axial slices, thickness = 1 mm, no gap, repetition time (TR)/echo time (TE) = 1000 ms/20 ms, flip angle = 90°, and single-shot gradient-echo echo-planar imaging (EPI). Anatomical images were obtained by turbo spin-echo with scanning parameters of TR = 2560 ms, TE = 38 ms, echo train length = 7, number of excitations = 1, FOV = 30 × 30 mm^2^, matrix size = 320 × 320, and slice thickness = 1 mm.

### Data analysis

The fMRI imaging data were first preprocessed using analysis of functional neuroimaging (AFNI) toolbox (http://afni.nimh.nih.gov). Data acquired in the first 10 volumes were discarded as the MRI signal has not yet reached a steady state. The brain EPI image of one control rat was selected as the template, the spatial dimension of which was used to normalize the brain images of all animals to reduce variations in brain size. Additional data preprocessing including despike, slice timing, head motion correction, linear detrend, and in-plane spatial smooth with a full width-half maximum (FWHM) kernel at 0.9375 mm were performed. As the raw spatial resolution of the EPI data was 0.46875 mm, the FWHM of the spatial filter was determined to be the doubled pixel size [17]. Although regression analyses for white matter (WM) and cerebrospinal fluid (CSF) are usually employed when processing human rs-fMRI data, WM/CSF regressions did not improve the detection specificity of neural activity in rodent rs-fMRI studies due to the very different anatomy between rats and human [18]. As a result, nuisance removal, such as regressing WM/CSF-derived signals, was not applied in this study. After preprocessing, pixel-by-pixel fALFF map were calculated by using AFNI toolbox. The fALFF was defined as the sum of amplitude across 0.01–0.1 Hz divided by that across the entire frequency range [6]. The regions-of-interest (ROIs) of the cingulate cortex [19], hippocampus [20], insula [21], somatosensory [22], striatum [22, 23], thalamus [24], and whole brain were manually delineated based on the rat brain stereotactic atlas [25]. These regions were the key regions involved in diabetes-related abnormalities, based on previous clinical and animal studies.

### Statistical analysis

All statistical analyses were performed using MATLAB (Version: R2020a) and visualized using Excel. The measurements were summarized as mean and standard deviation. One-way analysis of variance tests with repeated measures were performed on the weight and plasma glucose concentration data in each group to compare the differences across different time points. To assess region-specific differences between the groups, Student’s t-tests were applied to pairs of animal groups of the fALFF values. Statistical significance was set at P < 0.05.

## Results

**Fig. 1:**
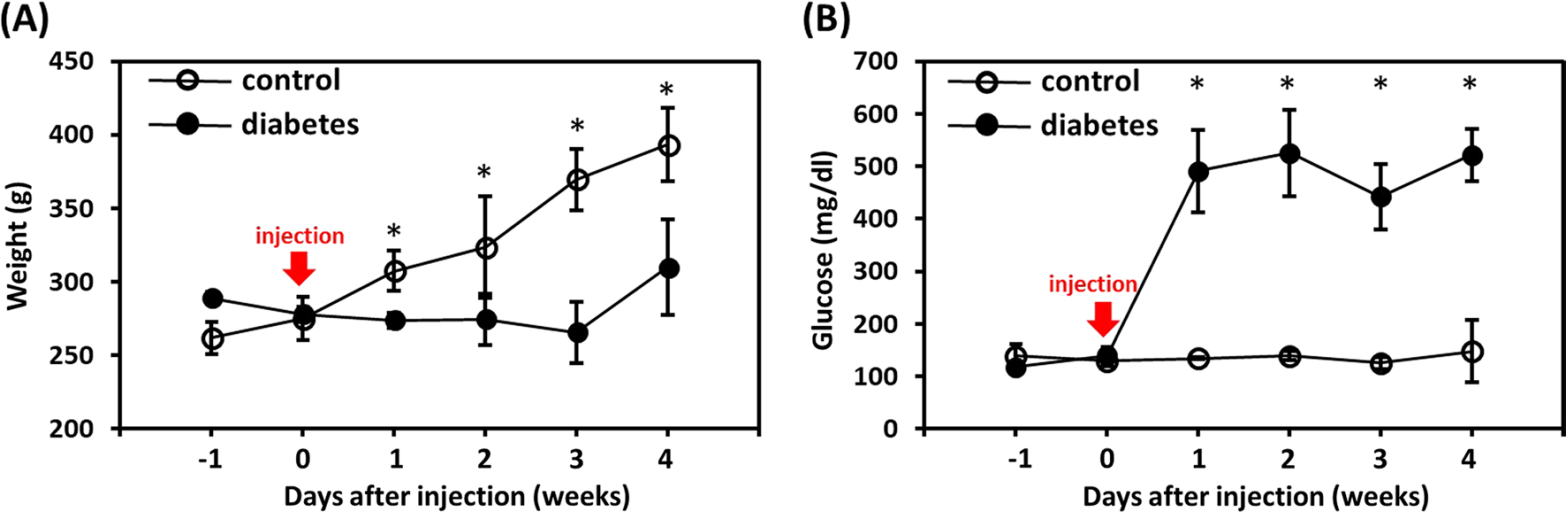
Weekly **a** weight and **b** plasma glucose level measurements for animals in the control and diabetes groups. *The difference between the two groups is statistically significant

The weights and plasma glucose concentrations in the animals are shown in Fig. 1a, b, respectively. Rats in the control group showed increased body weight over time following solvent administration (P < 0.05). Rats in the diabetes group showed early weight loss after STZ administration, followed by a slight increase in weight. No changes in plasma glucose concentration over time were present in the control group (P = 0.73). In contrast, the plasma glucose level significantly increased in the diabetes group at 1 week after STZ administration (P < 0.05), suggesting the development of diabetes in these animals. The hyperglycemia persisted until 4 weeks after STZ administration.

**Fig. 2:**
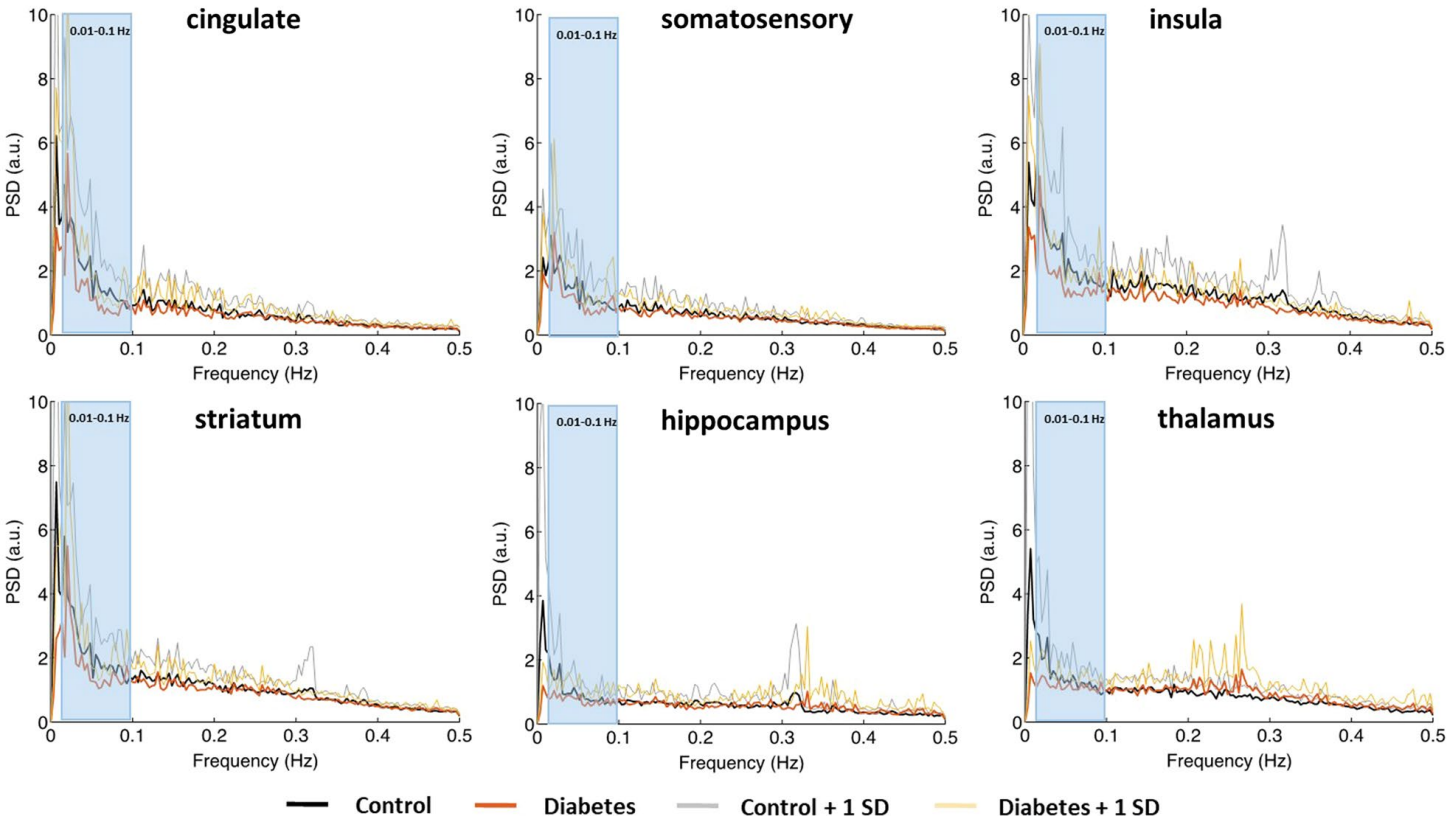
Power spectral density (PSD) in different brain regions between control and diabetes groups. The shaded regions indicate the frequency range (0.01–0.1 Hz) for calculating fractional amplitude of low-frequency fluctuation. SD, standard deviation within each group; a.u., arbitrary unit

**Fig. 3:**
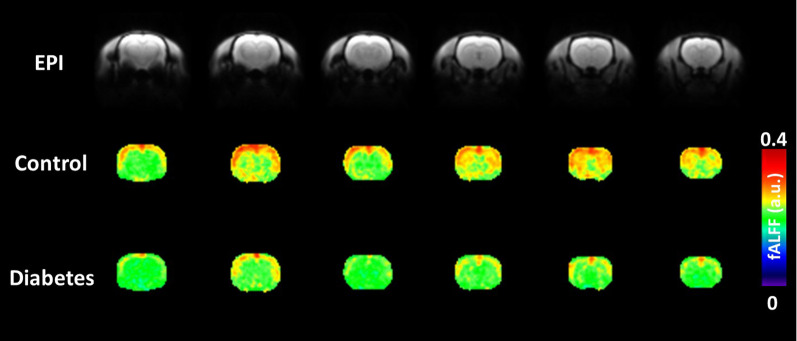
Fractional amplitude of low-frequency fluctuation (fALFF) maps in the control and diabetes subgroups. The data of animals from each group were averaged for display, and six representative brain sections are shown. EPI, echo-planar imaging

Power spectral density (PSD) in different brain regions between control and diabetes groups are shown in Fig. 2. Notably, compared to the control rats, the diabetic rats expressed lower PSD, primarily within the low-frequency region, in various brain regions. Quantitative fALFF maps stratified by group are shown in Fig. 3. The cortex demonstrated a relatively high fALFF than the other brain areas, suggesting high neural activity in the cortex. Visual inspection suggested that group-related differences in fALFF were homogeneous across brain regions, as rats with diabetes had lower fALFF compared to their control counterparts, in accordance with the results of power spectrums.

**Fig. 4:**
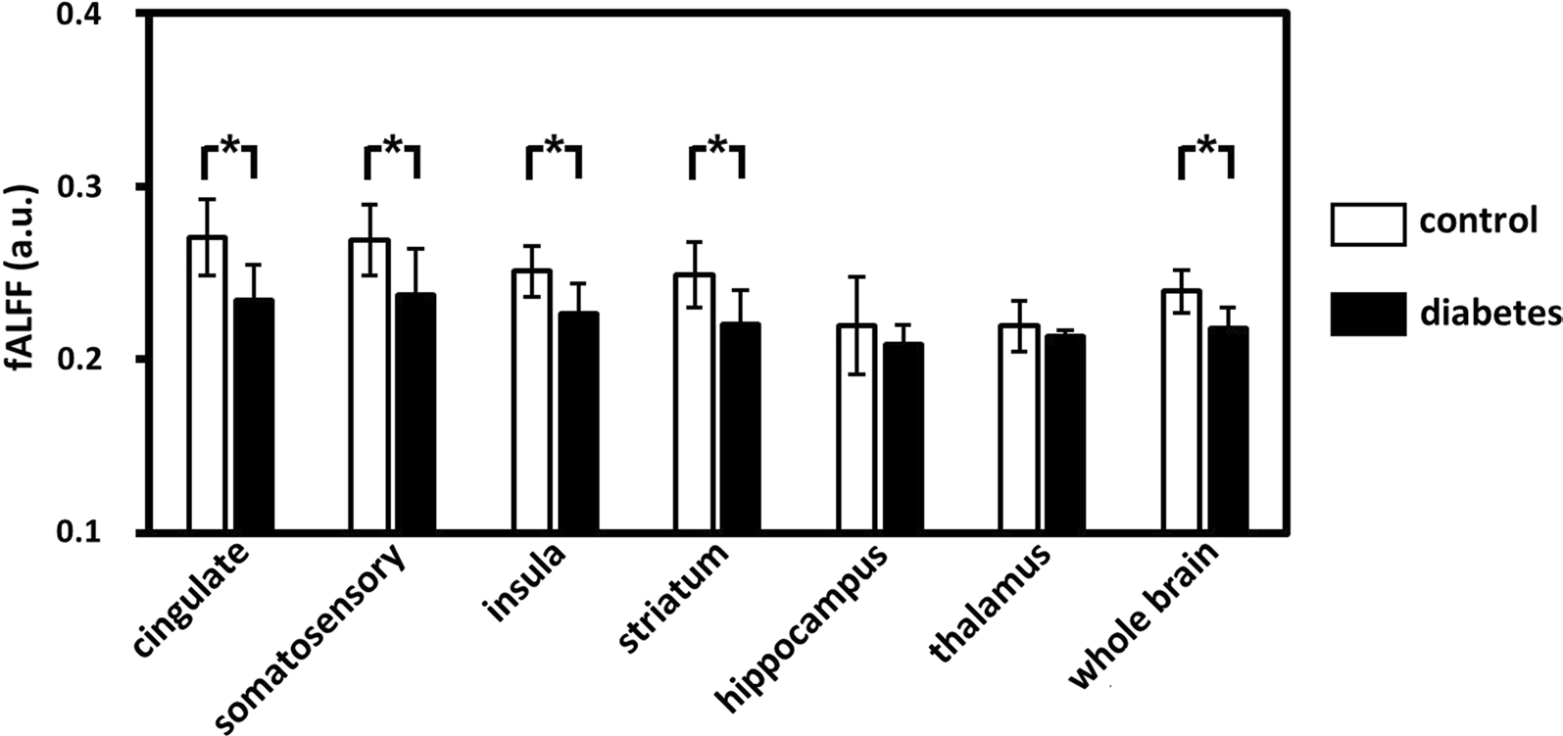
Mean fractional amplitude of low-frequency fluctuation (fALFF) of the regions of interest averaged across rats in the control and diabetes groups. *P < 0.05; a.u.: arbitrary unit

Figure 4 plots the results of the ROI analysis of the spatial distribution of the fALFF at the group level. The whole-brain fALFF was significantly lower in rats in the diabetes group compared to that in the control group (P < 0.05). In terms of regional analyses, a two-sample t-test showed significant differences between the two groups in brain regions including the cingulate cortex, somatosensory cortex, insula, and striatum (all P < 0.05), with the diabetes group showing the significantly lower fALFF values than those in the control group. None of the regions showed the opposite result, i.e., lower fALFF in the control group compared to the diabetes group.

## Discussion

The results of this study demonstrated for the first time decreased neural activity in specific brain regions, as assessed by the fALFF index, in diabetic animals compared to those in the control group even under anesthetic conditions. Aberrant neural activities were primarily observed in the cingulate cortex, somatosensory cortex, insula, and striatum. These findings provide new information on tracking neural abnormalities in diabetic animals using rs-fMRI techniques.

Although the pathogenesis of brain dysfunction in diabetes is not fully understood, research has showed that hyperglycemia leads to both metabolic and vascular disturbances in widespread brain regions, resulting in compromised brain function [26, 27]. Among these regions, striatum is gaining increased attention [23]. Previous studies have suggested that the striatum play a significant role in mood symptoms, and abnormalities in the striatum are correlated with depression disorder [28, 29]. From an epidemic perspective, diabetes has been associated with an increased prevalence of depression [30]. Consistent with previous study findings, the pathophysiological changes in the striatum found in the present study tentatively suggest that fALFF analyzed using rs-fMRI techniques could serve as an imaging biomarker to measure the aberrant neural activity in an animal model of diabetes. To some extent, the fALFF strategy may have the potential to further understand diabetes-related depression in an animal model. Additional research is necessary to explore this possibility.

Another key region involved in diabetes-related abnormalities is the cingulate cortex, which exhibited increased neural activity in patients with diabetes compared to control subjects [19]. However, this is in contrast with the finding of decreased fALFF in our diabetic animals. A clear explanation for this discrepancy is lacking, but may be related to the fact that human patients often have various comorbidities such as hypertension and obesity, which have also been shown to alter brain function [31, 32]. Using the diffusion tensor imaging (DTI) technique, Frokjaer et al. demonstrated diabetes-induced microstructural damage in the cingulate regions as evidence by the reduction in fractional anisotropy (FA) in patients with diabetes [33]. FA indicates the organization of fibers, such that a lower FA value is associated with decreased neural activity [34]. Consistent with the above study findings, the decreased fALFF in the cingulate region observed in the present study may reflect the actual effect of diabetes. Collectively, our findings could provide directions for future studies in this field.

Brain damage in a rat model of STZ-induced diabetes has been assessed using various techniques, which have shown that the hippocampus is the region most sensitive to hyperglycemia compared to other brain regions [35, 36]. Moreover, STZ injection led to a reduction in the number of pyramidal neurons in the rat hippocampus [20]. Disappointingly, we did not observe a difference in hippocampal neural activity between control and STZ-induced diabetic rats. This inconsistency may be partially attributed to the limitation that we only performed rs-fMRI at 4 weeks after STZ administration and not later. This time span was suggested by other MRI studies [22, 37], which showed considerable brain alterations 4 weeks after STZ administration. The hippocampus is involved in learning and memory, and impaired cognitive function is one of the major complications in patients with diabetes [38]. In STZ-induced animals, cognitive impairment is prominent at 12 weeks after diabetes onset [39]. Therefore, cognitive ability may function integrally at the early onset of diabetes in rats, resulting in the unaltered neural activity in the hippocampus observed in this study. However, as cognitive function was not measured and was beyond the scope of this study, further studies are needed to determine whether changes in cognitive function are associated with altered brain activity in the hippocampus of STZ-induced diabetic animals.

The use of anesthetics is an essential step in performing rodent MRI experiments to reduce motion and prevent stress. The commonly used anesthetic in rodent fMRI is isoflurane, owing to its advantages of maintaining a stable anesthetic plane for a longer time and rapid regaining of consciousness after anesthesia discontinuation [40, 41]. Although cerebral activity under isoflurane anesthesia is significantly suppressed [42], isoflurane acts as a vasodilator, which allows hemodynamic fluctuations linked to local neural activity to effectively spread through a larger cortical area [43]. Additionally, the spectral power of the cortex was enhanced under isoflurane anesthesia [44], suggesting the potential beneficial effect of isoflurane on boosting fALFF contrast. While this network structure is significantly distinct from the awake status, this phenomenon may be considered as a unique characteristic in isoflurane anesthesia for rodent rs-fMRI studies. Other potential anesthetics for rodent rs-fMRI studies include propofol and urethane, the induced neural activity patterns have many similarities to that in the awake condition [44]. As the effect of anesthetics on neural activity patterns is not always known and different pharmacological actions of different anesthetic regimes may induce varied brain responses, further diabetic animal rs-fMRI studies under different anesthetics may improve the data quality, comparability, and interpretation.

The results of this study must be interpreted with caution due to its limitations. First, as we only used one strain (Sprague–Dawley) of male rats, the results presented here may not be generalizable to other animals. Different strains of animals reportedly show different patterns of neural circuits [45]. Thus, additional, similar studies using different strains are needed to evaluate strain-dependent responses. Second, although they share the same features of persistent hyperglycemia, type 1 and type 2 diabetes have distinct pathophysiological effects and therefore exhibit different patterns of cerebral abnormalities [46]. As this study included only animals with type 1 diabetes, comparisons across different types of diabetes are needed. Third, the major disadvantage of rs-fMRI based on the EPI sequence is the signal loss due to the susceptibility artifacts, particularly in the brain region of the amygdala [47]. The amygdala is the key node for integrating information on stress and emotion regulation, and the disruption of these functions has been associated with neurocognitive deficits in diabetic patients [48, 49]. As each imaging modality has specific strengths and limitations, various techniques can be integrated to allow better estimation of the physiological characteristics of brain function in diabetes. Fourth, there are also many other analysis methods used in rs-fMRI, such as functional connectivity and independent component analyses, and only the fALFF approach was used in this study. To better apply the rs-fMRI to an animal model of diabetes, an evaluation of the same dataset analyzed with different strategies would be the paramount direction. Finally, we determined our sample size based on previous studies of animals with diabetes [12, 50, 51]. Our sample size was small; therefore, the significance was not maintained after multiple comparisons. Given the exploratory nature of this study, we compared fALFF between groups in terms of ROI analysis. The primary advantage of ROI analysis is that it controls for Type I errors [52]. Moreover, the fALFF in the whole brain was significantly different between groups, thereby increasing our confidence that fALFF is a powerful index to investigate the diabetes-related brain alterations. However, further studies with larger sample sizes are suggested for more robust statistical analysis, as well as for determining whether the brain function in hippocampus and thalamus are altered after the onset of diabetes.

In conclusion, this exploratory study is the first to investigate the effect of diabetes on neural activity using the fALFF approach in anesthetized rats. The results demonstrated significant reductions in neural activity in various brain regions of diabetic animals. These findings support the ability of the fALFF approach to differentiate healthy controls from diseased animals even under anesthesia to provide meaningful information regarding the neurological pathophysiology of diabetes using animal models.

## Data Availability

The datasets generated and/or analysed during the current study are not publicly available due to ethical issues but are available from the corresponding author on reasonable request.
